# Data on patient’s satisfaction from an emergency department: Developing strategies with the Multicriteria Satisfaction Analysis

**DOI:** 10.1016/j.dib.2018.10.041

**Published:** 2018-10-23

**Authors:** Panagiotis Manolitzas, Petros Kostagiolas, Evangelos Grigoroudis, George Intas, Pantelis Stergiannis

**Affiliations:** a Technical University of Crete School of Production Engineering and Management University Campus, Chania GR 73100, Greece; b Ionian University, Ioannou Theotoki 72 str., P.O. Box: 663, Corfu GR 49100, Greece; c Hellenic Open University, Parodos Aristotelous 18, Patra GR 26335, Greece

**Keywords:** Emergency department, Patient satisfaction, Decision making, MUSA (Multicriteria Satisfaction Analysis)

## Abstract

This article presents data that examine the patient׳s satisfaction from the services of an Emergency Department in Greece during the economic crisis. 490 questionnaires have been collected for the assessment of patient satisfaction by taking into account criteria like cleanliness, waiting room, access to the hospital, courtesy, friendliness and professional attitude of the emergency department staff, service processes and waiting times. In order to examine the satisfaction levels of the patients and moreover to design possible strategic actions we use the MUSA method.

**Specifications table**

**Table:** 

Subject area	*Healthcare Management-Public Administration*
More specific subject area	*Patient satisfaction*
Type of data	*Table, figure*
How data was acquired	*The sample was collected using a questionnaire. The data have been collected during the patient’s visit to the Emergency Department (ED) Ward.*
Data format	*Analyzed*
Experimental factors	*A pilot study was conducted using the questionnaire that had been developed. The doctors of the ED asked to express their opinion about how easy and understandable the questionnaire was and what adjustments they would propose to the research team to improve it.*
Experimental features	*In order to examine the services of the emergency department we used the MUSA method. Based on the results of the multiple criteria analysis we propose strategic actions for the ED based on patient’s perceptions.*
Data source location	*General Hospital of Nikaia-Emergency Department, Athens, Greece*
Data accessibility	*The Data are available with this article*

**Value of the data**

• The dataset can be used by other researchers in carrying out research in the area of patient satisfaction.
• The proposed questionnaire could be generalized for the assessment of the ED services in Greece and worldwide.
• Our data can be compared with other data across the globe for a comparative analysis.
• The analyzed data present which criteria are the most important for the patients in order to feel satisfied.
• Using the action diagrams of MUSA method we draw strategic actions for the Emergency Department.

## 1 Data

The research was conducted at the emergency department of the General Hospital of Nikaia, Greece during the period of April 2018. The scope of the survey was to collect data (*n* = 490 patients) from the Emergency Department in order to assess the satisfaction of the patients from the offered services. For the collection of the data we used a structured questionnaire that has been proposed by [1], for the evaluation of the Emergency Department Services.

The questionnaire is divided into two sections: (i) in the first section we collect information like demographics and (ii) in the second section we evaluate the patient׳s satisfaction by taking into account by 10 criteria: (1) Cleanliness, (2) Waiting room (layout, available chairs), (3) Access to the hospital, (4) Courtesy, friendliness, and professional attitude (physicians), (5) Responsiveness and personal care (physicians), (6) Courtesy, friendliness, and professional attitude (nurses), (7) Responsiveness and personal care (nurses), (8) Courtesy, friendliness, and professional attitude (administrative staff), (9) Service processes, (10) Waiting times. For the evaluation of the criteria a 5-point Likert scale has been used: dissatisfied, somehow dissatisfied, neither satisfied nor dissatisfied, somehow satisfied, satisfied.

## 2 Experimental design, materials, and methods

### 2.1 Method

The MUSA method is a multicriteria approach that has been developed in order to measure and analyze customer satisfaction [2], [3]. It approaches a research problem, *i.e.* patient satisfaction measurement, as an optimization one and goal programming techniques are used to solve it. Moreover, MUSA also applies a post optimality analysis phase in order to overcome potential problems related to model’s instability. The ultimate solution is attained as the average of the near optimal solutions of linear programming, which maximize the weights of the n satisfaction criteria [2], [4]. It should be noted that the main advantage of the MUSA method is that it fully considers the qualitative form of customer׳s judgements and preferences, as expressed in a customer satisfaction survey [4].

MUSA method provides a large set of outcomes like criteria weights, satisfaction indices, and action diagrams. Based on these results the organization has the advantage to have a clear view about the current state of the offered services and moreover to design effective strategies using the information that has been elucidated from customer׳s views.

### 2.2 MUSA method brief presentation

The MUSA method assesses global and partial satisfaction functions $Y^{*}$ and $X_{i}^{*}$ respectively, given customer׳s ordinal judgments Y and $X_{i}$ (for the *i*-th criterion). The assumption of an additive utility model is the main principle of the method, and it is represented by the following ordinal regression analysis equation:

(1) $${\tilde{Y}}^{*}=\sum_{i=1}^{n}b_{i}X_{i}^{*}-\sigma^{+}+\sigma^{-}$$

where ${\tilde{Y}}^{*}$ is the estimation of the global value function $Y^{*}$, n is the number of criteria, $b_{i}$ is a positive weight of the *i*-th criterion, $\sigma^{+}$ and $\sigma^{-}$ are the overestimation and the underestimation errors, respectively, and the value functions $Y^{*}$ and $X_{i}^{*}$ are normalized in the interval [0,100].

In this context, the customer satisfaction measurement problem may be formulated as an optimization problem using goal programming techniques, and thus, the estimation model can be written in an LP formulation, as follows:

(2) $$\{\begin{array}{c} \left[\min\right]F=\sum_{j=1}^{M}\sigma_{j}^{+}+\sigma_{j}^{-}\\ \mathrm{subject}\;\mathrm{to}\\ \sum_{i=1}^{n}\sum_{k=1}^{x_{i}^{j}-1}w_{ik}-\sum_{m=1}^{y^{j}-1}z_{m}-\sigma_{j}^{+}+\sigma_{j}^{-}=0\;\mathrm{for}\;j=1,2,\ldots ,M\\ \sum_{m=1}^{\alpha -1}z_{m}=100\\ \sum_{i=1}^{n}\sum_{k=1}^{\alpha_{i}-1}w_{ik}=100\\ z_{m},\;w_{ik},\;\sigma_{j}^{+},\;\sigma_{j}^{-}\;\forall \;m,i,j,k \end{array}$$

where M is the size of the customer sample, while $y^{j}$ and $x_{i}^{j}$ are the *j*-th level on which variables Y and $X_{i}$ are estimated (*i.e.* global and partial satisfaction judgments of the *j*-th customer). Furthermore, the following transformation equations are used for the decision variables of LP (2):

(3) $$\{\begin{array}{cc} z_{m}=y^{*m+1}-y^{*m} & \mathrm{for}\;m=1,2,...,\alpha -1\\ w_{ik}=b_{i}x_{i}^{*k+1}-b_{i}x_{i}^{*k} & \mathrm{for}\;\;\;k=1,2,...,\alpha_{i}-1\;\mathrm{and}\;i=1,2,...,n \end{array}$$

where $y^{*m}$ is the value of the $y^{m}$ satisfaction level, $x_{i}^{*k}$ is the value of the $x_{i}^{k}$ satisfaction level, and α and $\alpha_{i}$ are the number of global and partial satisfaction levels.

The MUSA method includes also a post optimality analysis stage in order to overcome the problem of model stability. The final solution is obtained as the average of the near optimal solutions of linear programming, which maximize the weights of the *n* satisfaction criteria [2].

### 2.3 Experimental design

**Fig. 1:**
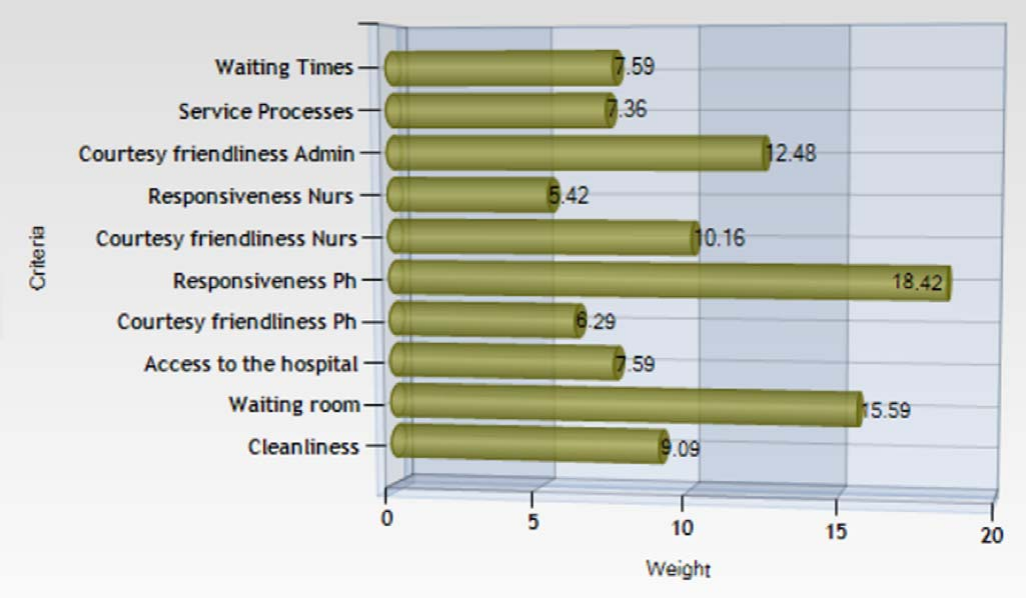
Criteria weights.

One of the most important result of the MUSA method is the weights of the criteria. The criteria weights reveal the relative importance according to a set of criteria or sub criteria that a group of customers (in our case the patients) assigns to the satisfaction dimensions. It should be noted that the weights are basically value tradeoffs among the selected criteria [2], [4], [5]. Based on Fig. 1 the most important criteria for the patients are the responsiveness of the physicians and the waiting room. On the other hand, lower levels of importance appear for the satisfaction dimensions like nurses’ responsiveness, courtesy and friendliness of the physicians as well as for the service processes.

**Fig. 2:**
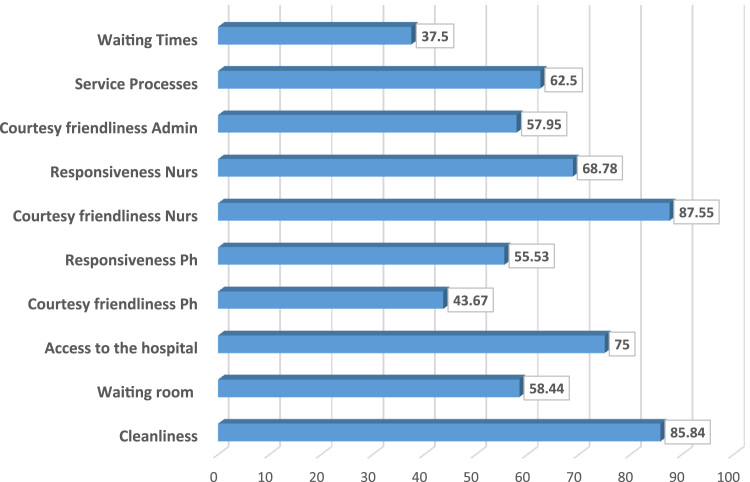
Satisfaction indices.

Beside the criteria weights, the MUSA method provides a set of average satisfaction indices. These average indices show, in the range 0–100, the level of global or single criterion customer satisfaction; they may be considered as the basic average performance indicators (globally or per criteria) for the organization [6]. A brief analysis of the Fig. 2 reveals that high levels of satisfaction are observed in criteria like cleanliness, courtesy and friendliness of the nurses. On the other hand lower levels of satisfaction are observed in criteria like courtesy and friendliness of physicians and waiting times.

Combing the results that have been derived by the MUSA methodology (criteria weights, average satisfaction indices) the methodology produces action diagrams which are similar to SWOT [4], [6], [7], [8], [9], [10], [11], [12] analysis and may present the strong and weak points of the emergency department, indicating which satisfaction dimensions should be improved.

**Fig. 3:**
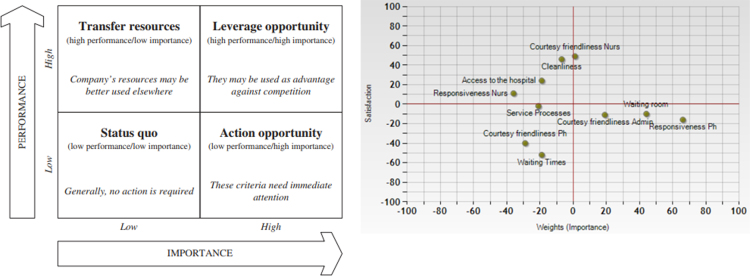
Action diagram.

The action diagram (Fig. 3) is divided into four quadrants: [1], [4], [6]: (i) *Status quo* (low performance and low importance): no action is required, given that these satisfaction dimensions are not considered as important by the customers, (ii) *Leverage opportunity* (high performance/high importance): This area can be used as advantage against competition, (iii) *Transfer resources* (high performance/low importance): Regarding the particular satisfaction dimension, company׳s resources may be better used elsewhere (*i.e.* improvement of satisfaction dimensions located in the action opportunity quadrant), (iv) *Action opportunity* (low performance/high importance): These are the criteria that need attention; improvement efforts should be focused on these, in order to increase the global customer satisfaction level. Through the action diagram (Fig. 2) it is clear that the strategic advantage of the ED department is the courtesy and friendliness of the nurses. Analysing the area of ‘action opportunity’ MUSA method reveals the weaknesses of the ED. The weaknesses of the ED has to do with the waiting room, the courtesy and friendliness of the administrative staff and the responsiveness of the physicians. At this case it is obligatory for the emergency department to take immediate actions of improvement. Criteria like Cleanliness, access to hospital and nurses responsiveness that belong to the upper left quadrant indicating that these criteria can be used as advantage against competition. The criteria that belong to the lower right quadrant (service processes, waiting times, courtesy and friendliness of physicians) ‘*status quo*’ area are the threats [10] of the ED because the satisfaction indices are low but their importance may be increased in the future.

It should be noted that the proposed data can be analysed by using the new methodological approach of MUSA-INT, giving the benefit to the researchers to take into account the positive and negative interactions among the criteria [13].
