# Supplementary material for: Data on patient’s satisfaction from an emergency department: Developing strategies with the Multicriteria Satisfaction Analysis
Source: Data Brief. 2018 Oct 23;21:956–61. doi: 10.1016/j.dib.2018.10.041 (PMC6222085; doi:10.1016/j.dib.2018.10.041)
Supplement: Supplementary file 1 — Supplementary material. [file mmc1.doc]

Conflict of Interest and Authorship Conformation Form

Please check the following as appropriate:

- All authors have participated in (a) conception and design, or analysis and interpretation of the data; (b) drafting the article or revising it critically for important intellectual content; and (c) approval of the final version.
- This manuscript has not been submitted to, nor is under review at, another journal or other publishing venue.
- The authors have no affiliation with any organization with a direct or indirect financial interest in the subject matter discussed in the manuscript

Author’s name Affiliation

Panagiotis Manolitzas, PhD Technical University of Crete

Petros Kostagiolas, PhD Ionian University

Evangelos Grigoroudis,PhD Technical University of Crete

George Intas Hellenic Open University

Pantelis Stergiannis Hellenic Open University
